# Operational analysis of school-based delivery models to vaccinate children against influenza

**DOI:** 10.1080/20476965.2020.1754733

**Published:** 2020-04-22

**Authors:** Luca Grieco, Mariya Melnychuk, Angus Ramsay, Abigail Baim-Lance, Simon Turner, Andrew Wilshere, Naomi Fulop, Steve Morris, Martin Utley

**Affiliations:** a Clinical Operational Research Unit, University College London, London, UK; b Department of Applied Health Research, University College London, London, UK; c Departmento De Economía De La Empresa, Economía Aplicada II Y Fundamentos De Análisis Económico, Universidad Rey Juan Carlos, Madrid, Spain; d Institute for Implementation Science in Population Health, City University of New York Graduate School of Public Health and Health Policy, New York, NY, USA; e School of Management, University of Los Andes, Bogotá, Colombia

**Keywords:** Flu vaccination, simulation, cost analysis

## Abstract

Large-scale immunisation programmes against seasonal influenza are characterised by logistical challenges related to the need for vaccinating large cohorts of people in a short amount of time. Careful operational planning of resources is essential for a successful implementation of such programmes. We focused on the process of child vaccination in schools and analysed the staffing and workflow aspects of a school-aged children vaccination programme in England. Our objectives were to document vaccination processes and analyse times and costs associated with different models deployed across England. We collected data through direct non-participatory observations. Statistical data analysis enabled us to identify potential factors influencing vaccine delivery time and informed the development of a tool to simulate vaccination sessions. Using this tool, we carried out scenario analyses and explored trade-offs between session times and costs in different settings. Our work ultimately supported the local implementation of school-based vaccination.

## 1. Introduction

Until 2012, in the United Kingdom, annual vaccination against seasonal influenza had been routinely offered only to “at-risk” groups including older people (>65 years of age), pregnant women, people with asthma. In July 2012, the UK Department of Health’s Joint Committee for Vaccination and Immunisation (JCVI) recommended that healthy school-aged children be vaccinated as well, using a nasal spray vaccine, to reduce the impact of influenza in children and avert many cases of severe influenza or influenza-related deaths occurring among those with clinical risk factors and older adults (Joint Commission on Vaccination and Immunisation, 2012).

Based on these recommendations, in 2013 the UK Department of Health and Public Health England commissioned seven areas in England to conduct a pilot implementation of vaccination among school-aged children to develop and test operational strategies to deliver the vaccine. Most of the pilot areas adopted school-based programmes, with teams of school nurses and other National Health Service (NHS) staff visiting primary schools to vaccinate children, whereas one area offered vaccination via local pharmacies. These areas delivered vaccination to primary school children aged 4 to 11 (i.e., from reception to school year 6) in 2013/14 and 2014/15 school seasons. In 2015/16, this vaccination programme underwent a national extension whereby children aged 5 and 6 (i.e., school years 1 and 2) were offered school-based vaccination all over England, with pilot areas continuing to offer school-based vaccination to children aged 4 to 11.

Existing literature on this topic is mainly descriptive of the US context and focused on the delivery of vaccination programmes at the regional level, without being informed by the use of organisational theory (Borja et al., 2009; Carpenter et al., 2007; Cho et al., 2011; Deuson et al., 1999; Effler et al., 2010; Hull & Ambrose, 2011; Klaiman et al., 2014; Schieber et al., 2012; Schmier et al., 2008; Tran et al., 2010). Therefore, evidence directly relevant to the UK was required to provide recommendations on the roll-out of this programme at the national level as well as for the planning, communication, and operational arrangements at the local level, accounting for specific settings on aspects such as funding models, workforce roles and regulations, and policy context (Perman et al., 2017). A multi-disciplinary team of operational, health economic, organisational, and attitudinal researchers was commissioned by the UK Department of Health Policy Research Programme to conduct an evaluation of the operational processes and implementation strategies to deliver this programme. One of the foci of our project was logistics of the vaccine delivery and administration, which constitutes a major challenge for this programme if extended at the national scale: up to 24,000 schools (depending on age range targeted) would need to be visited to immunise children within a period of 3 months, with the vaccine being characterised by very limited shelf life and subject to strict rules on maintaining refrigeration throughout storage and distribution. Therefore, management of vaccine supply chain and proper planning of vaccine administration schedules and workforce are essential for a successful implementation of the programme.

In this paper, we report on our analysis of the different operating models adopted by pilot areas – and then by providers nationally – to deliver influenza vaccination in schools. The objectives of this analysis were: (1) to observe and document how vaccines were delivered in schools by different providers; (2) to inform policymakers about vaccination session duration and cost associated with different operating models and about factors potentially affecting efficiency; (3) to develop data modelling tools and approaches to support operational planning of school visits.

We analysed observational data collected during the vaccination campaign to identify and test factors potentially influencing time to deliver the vaccine. Then, we developed a simulation tool to explore trade-offs between vaccination session times and monetary costs incurred, for different staff mixes and settings.

Note that in our context, given the need for vaccination teams to cover all schools within a rather short time window, time has, in general, a greater importance than monetary costs in determining the best team configuration. However, the importance of such metrics relative to each other may vary depending on the specific organisation’s needs, on the available workforce and on the different points in time when vaccinations are planned during a campaign. For this reason, our approach focused on presenting time/cost trade-offs for different operational models, rather than determining optimal solutions.

Research has already been published about the development of simulation tools for quantitative evaluation of vaccination processes. Aaby et al. (2006b) used simulation to model the process of vaccine delivery in case of outbreak of a contagious disease and supported capacity planning in the USA. Washington (2009) also estimated costs associated with vaccine delivery in such emergency circumstances. Other groups (Hupert et al., 2009, Aaby et al. 2006a; Kilianski et al., 2014; Spitzer et al., 2007) used similar modelling frameworks to integrate results obtained from mass prophylaxis exercises organised by public health departments in the USA. However, school-based vaccination processes are quite different from mass vaccination processes: for instance, the former are characterised by a much lower level of urgency, leading to markedly different demand patterns (including the possibility of scheduling vaccination sessions in advance and the timing of service request by patients). Therefore, we implemented a novel, bespoke simulation tool to carry out the second part of this work.

## 2. Methods

### 2.1. Data collection

During 2014/2015 and 2015/2016 flu vaccination seasons, we visited 16 schools (8 per season), spanning 7 areas across England and 8 different vaccination providers, to carry out observations on their vaccination days. Visits took place between November and January of each season. On the vaccination day, we met the provider’s vaccination team at the school and followed them throughout the vaccine delivery process without interfering with their operations. We observed and recorded details on the setting up procedures and collected quantitative data about the vaccination process: i) process flows, ii) types of staff involved and their tasks, iii) service times at each stage for each child.

Further input for our quantitative analyses included: school-level vaccine uptake rates provided by Public Health England; publicly available data on unit costs for healthcare, with staff hourly rates being £42/hour for nurses, £21/hour for healthcare assistants and £10/hour for administrative staff members (Personal Social Services Research Unit, 2014).

We used the following measures to compare the delivery models observed:

- *total time* to deliver the vaccine to an entire school (excluding breaks);
- *total cost* for staff to deliver the vaccine to an entire school (obtained by multiplying total time by staff hourly rates, and summing up for all staff deployed);
- *cost/child ratio*, obtained by dividing the total cost by the cohort size;
- *time/child ratio*, obtained by dividing the total time by the cohort size;
- average staff *utilisation rate* (i.e., proportion of total time staff members are busy rather than idle).

### 2.2. Regression analysis

Based on insights obtained during observations, we identified factors that might affect time to deliver the vaccine. We tested some of these factors (namely, children’s age group and child-to-staff ratio) through linear regression analysis using Stata/MP software package, the dependent variable being the activity time per child in minutes for a staff member and the explanatory variables being the age group, the number of staff members deployed and the child-to-staff ratio (for each staff type).

### 2.3. Modelling framework

Unbiased comparison of overall time and cost associated with different vaccine delivery models, or with different configurations of the same model structure, would require using a set of consistent parameters in the analysis. For instance, in order to compare two delivery models with the same flow of children but with two different staff mixes, we would need to consider two teams with similar (staff type-specific) activity times per child vaccinating two similar cohorts of children. The relatively small number of schools visited did not provide us with an exhaustive list of cases to carry out a systematic comparison solely based on gathered data. Therefore, we developed a modelling framework to represent and analyse vaccination sessions and used it as a basis to implement a simulation tool to estimate overall time and cost associated with different delivery models. This also enabled us to estimate measures of interest (namely, staff utilisation rates) not quantifiable from our observational data.

Our modelling framework is based on results from queueing theory. Particularly, a vaccine delivery model is represented using a “queueing network” (Lazowska, 1984). Each activity (a “node” of the network) is characterised by a queue (a group of children waiting to be served) and one or more servers (staff members carrying out that activity). Once a child has been delivered an activity, they go to one of the following activities in the network based on the specific model adopted by the provider. Each activity is characterised by the type and number of staff members (each treating one child at a time); the type of activity; and the average (staff type-specific) time needed to deliver it to each child.

Based on the above framework, we developed a piece of software enabling the simulation of a vaccine delivery session. Our simulator takes the following types of information as input: all possible pathways children can undergo across the network; attributes for each node of the network as specified above (i.e., type of staff, type of activity, number of staff, average time per child).

We considered the following outputs from the simulator in order to compare different process layouts and staff mixes in terms of associated time and cost:

- time to vaccinate a class of children, i.e., time elapsing from when the vaccination session begins to when all children have undergone the entire process;
- staff utilisation rate per class, i.e., proportion of total time staff members is busy rather than waiting for the next child to arrive from the previous activity.

The above outputs constitute building blocks to estimate our measures of interest related to an entire school:

- time to vaccinate the entire school, given by the sum of times to vaccinate each class;
- staff utilisation rate, given by averaging the staff utilisation rate per class across all classes in the school;
- staff cost to vaccinate the entire school, obtained by multiplying the time to vaccinate the entire school by staff hourly rates and summing up for all staff deployed (note that we did not consider set up/pack up times, possible breaks, as well as additional office time).

**Figure 1.**
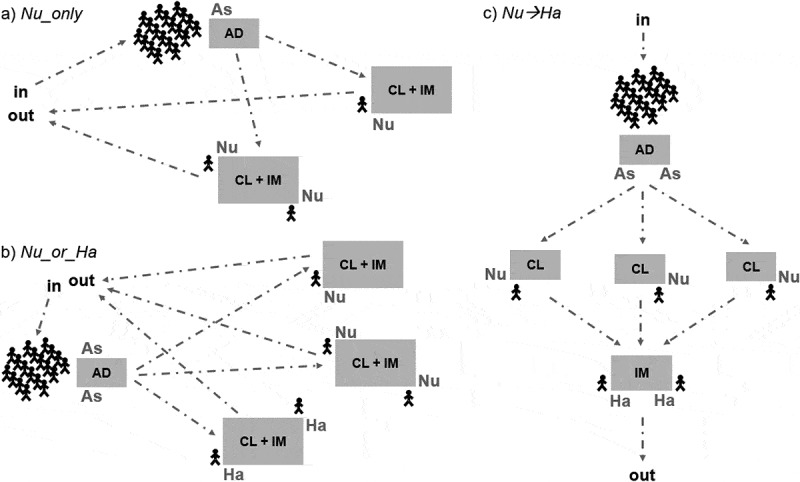
Examples of delivery models observed in seasons 2014/15 and 2015/16. Stylised humans represent children and dashed arrows represent possible flows of children through the vaccination process. Each box is a desk in the vaccination room that can be occupied by one or more staff members (As = administrative staff, Nu = nurse, Ha = healthcare assistant) carrying out their activities (AD = administrative tasks, CL = clinical checks, IM = immunisation). Following AD, children receive CL and then IM. We report here the three basic structures identified during our observations. (a) Nu_only: only nurses carry out the activity CL+IM; (b) Nu_or_Ha: either nurses or healthcare assistants carry out the activity CL+IM; (c) Nu→Ha: nurses carry out CL and then healthcare assistants carry out IM

We encoded our simulator using VBA programming language and embedded it within a Microsoft Excel spreadsheet, with a user-friendly interface. Users can define a list of activities and staff types to build networks similar to those depicted in Figure 1 and to compare scenarios characterised by different settings.

We let vaccination providers use this tool during the 2014/15 school season and refined our framework based on the feedback received. Such feedback was mainly qualitative and focused on the layout of the Microsoft Excel spreadsheet in view of a possible adoption of the tool by providers; however, it also enabled us to verify that our modelling assumptions were reasonable and that the input parameters of the simulator reflected the actual range of choices falling under the control of the decision-maker.

Finally, in order to validate our simulator, we tested it against the collected observational data. For each of the 16 schools observed, we simulated the corresponding delivery model using school-specific parameters, namely the network, the staff mix, and the activity times per child. The latter were extracted from our data and were assumed to be log-normally distributed with mean and standard deviation estimated from the observed values. Results for each class were averaged across 100 runs and then summed up to obtain the time to vaccinate the entire school. We compared these results with the time to vaccinate the same school as observed during school visits (cleaned from any set up, pack up, and break times).

## 3. Results and discussion

### 3.1. Delivery models observed

Children were usually brought to the vaccination room by class. Therefore, for our analyses, we defined *vaccination session* the sub-process consisting of vaccinating a single class in a school.

During a vaccination session, children went through the following sequence of *activities*:

Step 1 *Administrative tasks (AD)* – Identity of the child is checked; a staff member makes sure a consent form signed by parents or carers has been completed for that child.

Step 2 *Clinical checks (CL)* – The child is interviewed to determine whether they can take the vaccine on the day, also based on parents’ responses on the consent form.

Step 3 *Immunisation (IM)* – Vaccine is administered to the child via nasal spray; a certificate is given to the child reporting that they have received the vaccine.

According to guidance by Public Health England (2015), children can receive the vaccine under two different types of prescription: (i) Patient Specific Directions (PSDs) and (ii) Patient Group Directions (PGDs). PSD enables a non-prescribing healthcare professional to administer a prescription-only medicine such as a vaccine. PGDs were introduced as a facilitative measure to allow non-prescribing healthcare professionals to take a decision to supply or administer such medicines without the patient needing to see a prescriber, subject to the non-prescribing professional having been assessed as competent to do so. In relation to this project, the observed differences between the two types of prescriptions were reflected in staff members being allowed to perform either clinical checks or vaccine administration, or both. In particular, healthcare assistants (staff members with a lower level of qualification than registered nurses) were allowed to carry out clinical checks (CL) only to children with a PSD prescription (and thus already thoroughly assessed by a GP or a nurse prescriber), but they were allowed to administer the vaccine (IM) to any child independently of their prescription (PSD or PGD). Note that, in comparison with PGD prescriptions, PSD prescriptions require additional upfront activities (e.g., collecting and processing prescriptions) implying longer office and/or prescriber time.

In general, different *staff types* were deployed for different activities (in agreement with PHE guidance as well as specific provider’s choices), each staff member serving one child at a time:

- *Administrative staff (As)* members carried out AD;
- *Nurses (Nu)* carried out either CL only, or both CL and IM at the same time;
- *Healthcare assistants (Ha)* carried out either IM only, or both CL and IM at the same time.

Vaccination teams always included at least two staff members administering vaccines (i.e., nurses and/or healthcare assistants) with at least one of them being a nurse (i.e., a qualified healthcare professional). Usually, one or two administrative staff members were also part of the team.

Figure 1 depicts examples of the delivery models observed in our visits. Following set up procedures by the vaccination team, children were usually brought to the vaccination room (i.e., a dedicated school room, such as a gym or a canteen) by class and were accompanied by a staff member of the school. While waiting to undergo clinical checks and immunisation, they went through administrative tasks (i.e., an administrative staff member checked their identity and gave them their consent forms). Based on their type of prescription, each child was addressed to the first available nurse or healthcare assistant. Every child (or a group of children) was sent back to their class as they received the vaccine. A new class was brought to the vaccination room after the previous class was cleared. Breaks lasting a few minutes (or even lunch breaks) could take place between two consecutive vaccination sessions. At the end of the whole vaccination process, the vaccination team waited for a period of time (varying from a few minutes to half an hour) before leaving the school, for safety reasons (e.g., to provide care to any child experiencing side effects to the vaccine).

In terms of layout, we grouped the 16 vaccine delivery models observed into three structures (summarised in Figure 1):

- *Nu_only* – only nurses carrying out clinical checks and immunisation as a single step;
- *Nu_or_Ha –* either nurses or healthcare assistants carrying out clinical checks and immunisation as a single step;
- *Nu→Ha* – nurses carrying out clinical checks followed by healthcare assistants carrying out immunisation.

Though with varying numbers of staff members, *Nu_only* model was observed in nine schools, *Nu_or_Ha* model was observed in five schools, *Nu→Ha* model was observed in four schools. Please note that combinations of the above models, either in parallel (at the same time) or separately for different year groups, were deployed in two schools.

### 3.2. Time/cost analysis of observed vaccine delivery models

**Figure 2.**
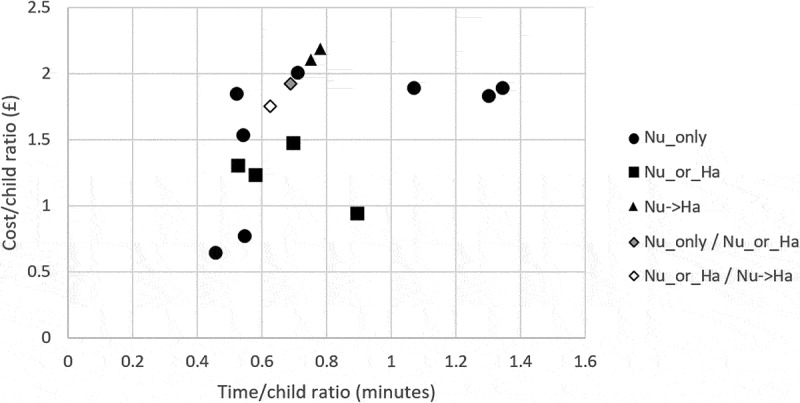
Time per child and staff cost per child associated with each of the 16 schools observed in seasons 2014/15 and 2015/16

Figure 2 summarises times and staff costs associated with the delivery models observed in seasons 2014/15 and 2015/16.

Staff cost per child in *Nu→Ha* model tended to be higher than in *Nu_only* model and *Nu_or_Ha* model (for similar time/child ratio), suggesting that configurations where each staff member carries out all activities might be more efficient than sequential processes. However, we shall note that in *Nu_or_Ha* model healthcare assistants can administer the vaccine to children, which is only allowed for children with a PSD prescription (i.e., already thoroughly assessed by GPs or nurse prescribers ahead of vaccination session). We were not able to account for the additional PSD-specific upfront activities in our study; therefore, all the following analyses mainly focus on assessing different configurations of each model, rather than comparing different models.

Albeit accounting for school sizes (we divided cost and time by the number of children vaccinated), variability is still high within each model type, particularly in *Nu_only* and *Nu_or_Ha*. We thus investigated whether additional factors might exist affecting time (and consequently cost) to deliver the vaccine in the three identified model structures. In particular, based on insights obtained during observations, we identified the following potential factors:

- *Children’s age group*. We considered two age groups: from reception to year 2 (R-2) and from year 3 to year 6 (3–6). The rationale for testing this factor is that staff might spend more time to deliver the service to younger children, for instance, because they need to keep reassuring them about the vaccine being painless.
- *Child-to-staff ratio*. We considered the number of staff members (for different staff types) deployed for a fixed cohort size. This factor is related to possible adaptive behaviours by staff members depending on their workload versus available time: for instance, when carrying out vaccination in a small school, staff members might have the possibility to dedicate more time to each child as opposed to vaccination in a big school.
- *Vaccination provider*. We observed very different ways to set up rooms for vaccination and to approach children during sessions across different providers. For instance, staff might be trained differently on how to deal with children, which might lead to different amounts of time to deliver the same exact activity. However, we could not include the vaccination provider as a factor in our analysis due to issues related to data samples (i.e., unbalanced representation of activities across different providers in the data).

Regression analysis of activity times was carried out independently for each of the three model structures identified. We did not consider administrative tasks and administrative staff in this analysis as the amount of data available was very limited (in most cases administrative tasks were delivered very quickly and/or while children were queueing so they were very unlikely to affect the total vaccination time).

Our results are reported in Table 1, with p-value threshold for statistical significance set to 0.01. The age group factor significantly influences time to deliver the vaccine. On average, it took longer to deliver vaccination activities to children in the age group R-2 than to those in age group 3–6 in 4 out of five cases tested. In terms of team size and child-to-staff ratio, statistically significant results were obtained in 2 out of 5 cases: CL&IM activity by nurse in *Nu_only* model and CL&IM activity by healthcare assistant in *Nu_or_Ha* model. In both cases, a higher child-to-staff ratio was associated with shorter activity time, suggesting the presence of adaptive behaviours among staff members. In the remaining cases, a lack of statistically significant association might be explained by the impossibility to compress activity times in some circumstances (e.g., in case of nurses and healthcare assistants working sequentially). However, contrasting evidence for the same activity (CL&IM) carried out by the same staff type (nurse) might suggest the presence of bias due to unbalanced sample sizes.

**Table 1.** Results from linear regression analysis (numbers rounded to two decimal places). Entries with p-values smaller than 0.01 are emphasised in bold

Basic model	*Nu_only*	*Nu_or_Ha*		*Nu→Ha*	
Activity type	CL&IM	CL&IM		CL	IM
Staff type	Nu	Nu	Ha	Nu	Ha
Age groups	Average activity times (minutes) and p-values				
Activity times for R-2	**1.83**	**2.16**	2.27	**1.46**	**1.92**
Activity times for 3-6	**1.61**	**1.76**	2.16	**0.98**	**1.36**
p-value	**0.00**	**0.00**	0.21	**0.00**	**0.00**
Staff members deployed	Linear regression coefficients and p-values				
Estimated variation (minutes) in activity time if one additional staff member deployed	**−0.26**	0.36	**−0.62**	3.80	0.32
p-value	**0.00**	0.16	**0.00**	0.60	0.11
Child-to-staff ratio	Linear regression coefficients and p-values				
Estimated variation (minutes) in activity time if one more child in the school	**−0.03**	0.09	**−0.22**	0.09	0.06
p-value	**0.00**	0.19	**0.00**	0.55	0.08

### 3.3. Simulation results

**Figure 3.**
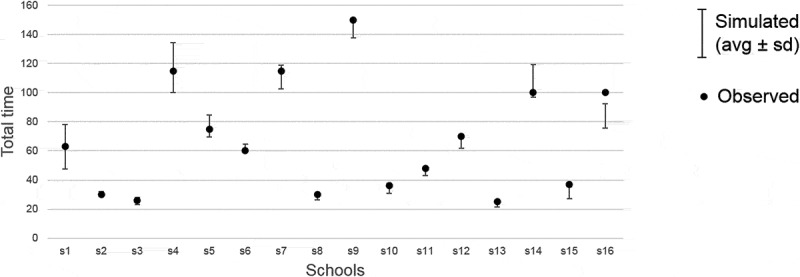
Results of simulation tool tests against observed data. For each school (s1 to s16), we report the actual observed time to complete the whole vaccination process (cleaned from any setup, pack up and break times) using dots. Results obtained by our simulation tool for each model are indicated by bars representing the average time across 100 simulation runs ± one standard deviation

**Figure 4.**
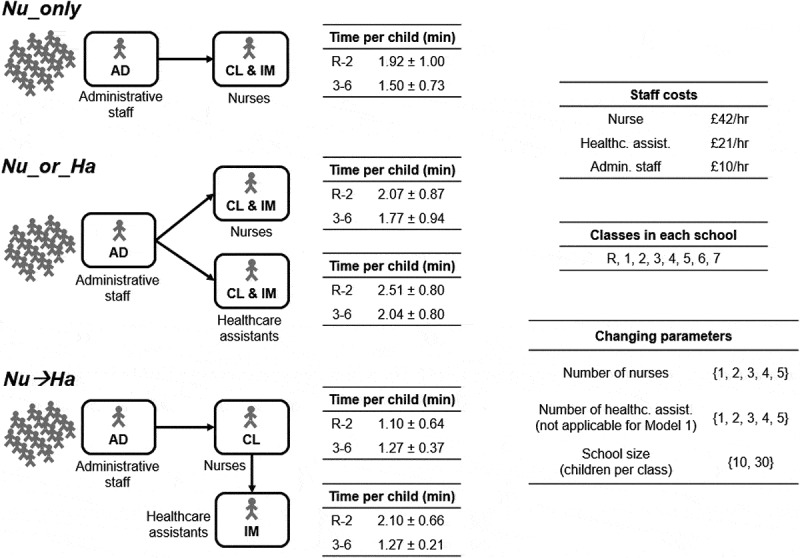
Simulation experiments. The figure summarises the sets of parameters used in simulations. Time per child was obtained from observed data by averaging corresponding activity times over all observed schools for each combination {activity, staff type, age group}. All possible combinations of changing parameters were used for each model, ensuring that, coherently with our observations, at least two staff members actually administering vaccines (i.e., nurses and/or healthcare assistants) are deployed, with at least one of them being a nurse

After verifying the consistency of the results obtained from our simulator with observed data (Figure 3), we used the simulator to study how staff mix variations can affect overall time and cost associated with the three vaccine delivery model structures identified above. The simulation experiments conducted are summarised in Figure 4. The range of values for each parameter was informed by observed data. In all the models, we assumed that administrative tasks are carried out while children are waiting in the initial queue (this is a very quick activity with no major influence on total time) and that two administrative staff members are deployed for the whole session length. Activity times for nurses and healthcare assistants were estimated from data and stratified into age groups (years R-2 vs years 3–6) due to our regression analysis results discussed above.

For each combination of parameters, time to clear the entire school and staff utilisation rate were estimated by averaging results across 100 simulation runs. Overall cost was estimated starting from estimated time to clear the entire school (cf. Methods). This analysis was conducted separately for each model structure.

**Figure 5.**
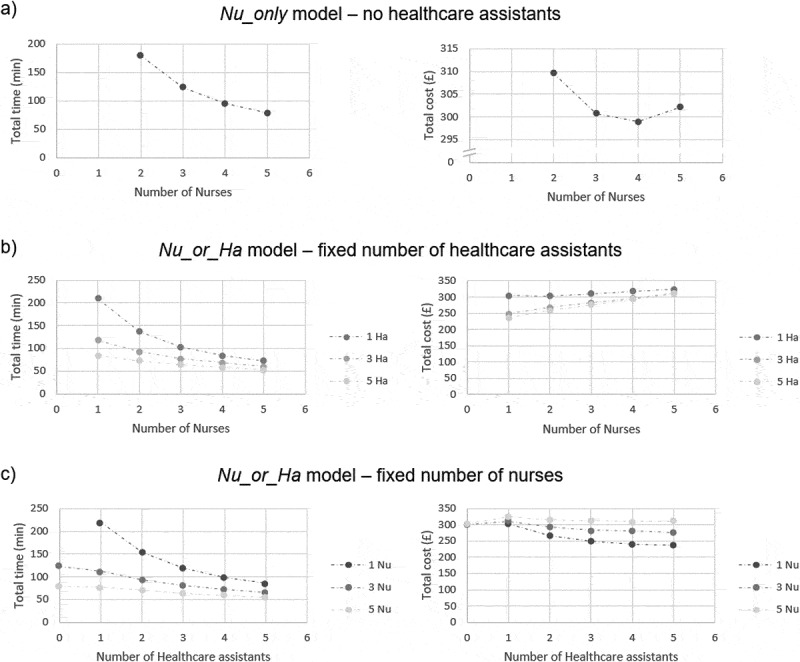
Simulation results for Nu_only and Nu_or_Ha models. Results were obtained using the parameters summarised in Figure 4. The graphs reported here correspond to the case of a hypothetical school with 30 children per class. Note: we emphasise the non-linear behaviour of the total cost in the Nu_only model (a – right side) by using a different scale for the y-axis

**Figure 6.**
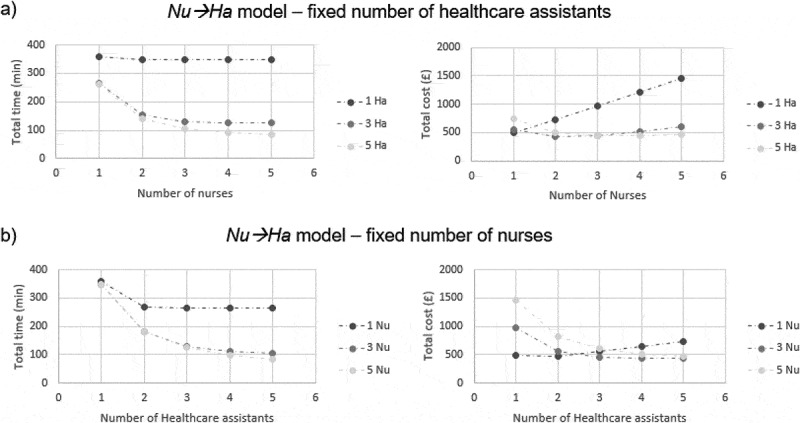
Simulation results for Nu→Ha model. Results were obtained using the parameters summarised in Figure 4. The graphs reported here correspond to the case of a school with 30 children per class

Figures 5 and 6 summarise our simulation results, using the parameters from Figure 4 and considering a hypothetical (big) school with 30 children per class. Except when explicitly specified below, model behaviour was the same for a different hypothetical (small) school with 10 children per class.

In *Nu_only* model, increasing the number of nurses reduces the time to vaccinate the entire school (Figure 5(a) – left-hand side) in a less-than-linear fashion due to randomness in operations, therefore leading to an increase in total nurse time and cost; when doubling the number of nurses, session length would only be halved if, by chance, all nurses finished their job exactly at the same time. However, a decrease in vaccination session length also translates into savings in terms of administrative staff time. Therefore, when a relatively low number of nurses (2 to 4) is deployed, the decrease in administrative staff cost due to shorter sessions is bigger than the increase in nurse cost due to randomness, so the total cost decreases as the number of nurses increases (Figure 5(a) – right-hand side). When the number of nurses deployed further increases, the increase in nurse cost due to randomness prevails and the total cost increases instead. A similar behaviour is observed in *Nu_or_Ha* model when only 1 healthcare assistant is deployed, whereas with a fixed number of 2 or more healthcare assistants the total cost always increases with the number of nurses (Figure 5(b) – right-hand side).

The effects of randomness on total cost are attenuated when additional staff members with lower hourly rates (i.e., healthcare assistants) are deployed (Figure 5(c) – right-hand side). However, in smaller schools (10 children per class, rather than 30), the total cost always increases with the number of any staff type (results not shown). Related to this, for instance, letting the number of healthcare assistants deployed alongside three nurses in the *Nu_or_Ha* model varies in {1, 2, 3, 4, 5} giving staff utilisation rates of {93%, 91%, 89%, 86%, 84%} in a big school and {83%, 77%, 72%, 68%, 65%} in a small school.

Together, these findings suggest that the marginal cost of deploying one or more staff member can be negated by the gain in time brought by using this additional staff member unless their wage is too high and/or their utilisation falls too low.

In *Nu→Ha* model, either by fixing the number of healthcare assistants and increasing the number of nurses, or by fixing the number of nurses and increasing the number of healthcare assistants, the time to vaccinate the entire school initially tends to decrease, but then seems to stabilise around a lower limit (Figure 6 – left-hand side). Figure 6 (right-hand side) also shows that, by increasing the number of any type of staff members, the total cost decreases up to a certain point, and then it increases. For instance, a reasonable trade-off between time minimisation and cost minimisation is found when the number of healthcare assistants is equal to the number of nurses plus 1. Therefore, coherently with the sequential nature of *Nu→Ha* model, our simulation results suggest that deploying additional staff members can be convenient, but only if a balance is kept between the number of nurses and of healthcare assistants.

## 4. Conclusions and limitations

Structural analysis of vaccine delivery models based on data systematically gathered during school-based observations allowed us to determine common and distinct features of processes adopted by different vaccination providers across England. This also helped us identify potential factors influencing time to deliver the vaccine. We tested such factors against collected quantitative data using linear regression analysis. Child age played a clear role in time associated with vaccination-related activities, with younger children requiring more time and attention. However, we encountered some difficulties in analysing the effects of other potential factors on activity times. Our analysis of observational data did not give clear results regarding the presence of workload-dependent adaptive behaviours in staff. Indeed, biases due to the presence of unbalanced sample sizes as well as the high variety of working arrangements observed across different providers made this analysis particularly challenging.

Insights from our data analysis were shared with the UK Department of Health and Social Care, Public Health England and NHS England for distribution to vaccination providers. In particular, these included information about observed variability of activity times and differences in activity times depending on child age or staff type (cf. Figure 4 – Time per child).

For a more comprehensive assessment of times and costs associated with the delivery models identified in the structural analysis, we developed a tool enabling the simulation of school-based vaccine delivery sessions. Simulations allowed us to explore trade-offs between session times and costs, for different staff mixes and settings. As activity times and staff costs used in our simulations were estimated from observed data, our quantitative results need to be considered in relation to the specific case analysed. However, our tool and approach could be easily adapted to other contexts and/or different mass immunisation programmes.

In our work, we focused on times and costs associated with the vaccination sessions in isolation and excluded some time/cost components: i) additional upfront activities carried out for children with PSD prescription; ii) set up and pack up procedures; iii) staff travelling from/to their base (either home or a health facility) and for which staff travel time presented a cost to providers. Regarding these activities, we were not able to gather data of enough detail for inclusion in our work. While this constituted a limitation to our approach, we believe our work could be easily extended to include those components when analysing a specific provider’s context, potentially enabling formal optimisation approaches.

This analysis of staffing and process layout options for school-based vaccination can help mitigate the difficulties of providing vaccination to a high number of schools in relatively short time windows. To this end, we shared our spreadsheet-embedded simulator with some vaccination providers involved in the pilot phase of the programme. Besides being a great opportunity to test our modelling assumption, this tool proved to be very useful for supporting decisions as some providers actually used it during planning of school visits in order to decide the process layout and staff mix to deploy on given days. The Microsoft Excel implementation of the simulator is freely available upon request.
